# Canine circovirus among dogs and cats in China: first identification in cats

**DOI:** 10.3389/fmicb.2023.1252272

**Published:** 2023-08-30

**Authors:** Xiangyu Xiao, Yan chao Li, Feng pei Xu, Xiangqi Hao, Shoujun Li, Pei Zhou

**Affiliations:** Guangdong Provincial Pet Engineering Technology Research Center, College of Veterinary Medicine, South China Agricultural University, Guangzhou, Guangdong, China

**Keywords:** canine circovirus, dogs and cats, high prevalence, genotypes, genetic recombination

## Abstract

Canine circovirus (CanineCV) is a virus associated with respiratory and digestive diseases in dogs and often occurs in coinfections with other pathogens, thereby aggravating the symptoms of infected dogs. CanineCV was first reported in the United States in 2012. Subsequently, it was reported among dogs in Europe, Asia, and South America. To investigate the prevalence of CanineCV in dogs in China, 331 dog samples were collected in this study. The PCR results showed that 9.06% (30/331, 95% CI = 6.2% ~ 12.7%) of the dog samples were CanineCV positive. CanineCV has also been detected in some carnivorous wild animals, indicating the potential risk of cross-species transmission of this virus. And, cats are also one of the most common pets in our daily lives, who is close contact with dogs. Thus, this study first investigated the prevalence of CanineCV in cats. The PCR results showed that 3.42% (14/409, 95% CI = 1.9% ~ 5.7%) of the cat samples were CanineCV positive. Moreover, 14 canine-derived CanineCV whole genomes and the first cat-derived CanineCV whole genome were obtained in this study. Rep and Cap are the major nonstructural proteins and structural proteins of CanineCV, respectively. In nucleic acid homology analyses, these 15 CanineCV strains showed a high degree of variation in Rep (85.9 ~ 99%) and Cap (85.6 ~ 100%). In phylogenetic analyses, the 15 CanineCV strains clustered into 3 different genotypes (genotypes 1, 3, and 4). Among them, the first cat-derived CanineCV belonged to CanineCV-3. In addition, 4 genetic recombination events were predicted in these 15 CanineCV strains, occurring in multiple regions of the genome. In conclusion, this study is the first to provide evidence of CanineCV infection in cats and successfully obtained the first whole genome of cat-derived CanineCV. The complex circulation and high prevalence of CanineCV among dogs and cats emphasize the importance of continuous monitoring of this virus in various animal species.

## 1. Introduction

Canine circovirus (CanineCV) is a member of the genus Circovirus, family Circoviridae (Kapoor et al., 2012). Circoviruses also include porcine circovirus (Meng, 2013; Palinski et al., 2017), goose circovirus (GoCV) (Guo et al., 2011), duck circovirus (DuCV) (Hong et al., 2018), and other bird viruses (Todd et al., 2001a,b; Hong et al., 2018; Gorbalenya et al., 2020). The genome of CanineCV is an approximately 2062–2064 nt DNA genome and consists mainly of 3 open reading frames (ORFs). ORF1 encodes the replicate protein (Rep) and ORF2 the capsid protein (Cap); ORF3 encodes a 105 amino acids (aa) of unknown function (Gomez-Betancur et al., 2023). Pathogenicity varies among circoviruses. Most members of the genus Circovirus infect susceptible animals and can induce lymphoid tissue damage, leading to immunosuppression and a range of serious secondary infections. For example, DuCV can cause immunosuppression, which increases the risk of coinfection with other pathogens (Yuan et al., 2022). Porcine circovirus 2 (PCV2) is related to postweaning multisystemic wasting disease syndrome (PMWS) (Allan and Ellis, 2000; Rakibuzzaman and Ramamoorthy, 2021). In addition, some circoviruses only cause subclinical infections in susceptible animals, such as porcine circovirus 1 (PCV1), which does not cause cytopathic effects, although it induces the production of serum antibodies in swine (Rakibuzzaman and Ramamoorthy, 2021). CanineCV is associated with respiratory diseases and diarrhea in dogs (Li et al., 2013; Decaro et al., 2014; Hsu et al., 2016; Anderson et al., 2017; Cruz et al., 2020; Dankaona et al., 2022), and CanineCV may also cause immunosuppression due to coinfection with other pathogens, leading to aggravation of clinical symptoms (Dowgier et al., 2017; Kotsias et al., 2019). As CanineCV could not effectively replicate on cells, its viral isolation and cultivation *in vitro* have been unsuccessful (Gomez-Betancur et al., 2023).

CanineCV was first identified among dogs in the United States in 2012 (Kapoor et al., 2012). Subsequently, it was reported among dogs in Europe, Asia, and South America (Gomez-Betancur et al., 2023). Hence, the natural host of CanineCV is dog. However, the presence of CanineCV has been detected in various wild animal species. Italian researchers have identified the presence of CanineCV in organ samples from wolves and badgers in indian (Zaccaria et al., 2016). British researchers have found that the nucleotide homology between fox circovirus and CanineCV is approximately 89% (Bexton et al., 2015). CanineCV was detected in both red and arctic foxes by Norwegian researchers, who further determined that the transmission of CanineCV within the arctic fox population can be traced back to 1996 (Urbani et al., 2021). The above findings demonstrate the risk of cross-species transmission of CanineCV, highlighting the need for further investigation into its prevalence among both wild and domestic animals. Herein, we describe an investigation of the prevalence of CanineCV in dogs conducted in Shanghai, Guangzhou, Foshan, and Zhengzhou in China, and this study also represents the first investigation of CanineCV prevalence in cats.

## 2. Materials and methods

### 2.1. Sample collection

From November 2021 to September 2022, a total of 740 samples were collected from 331 dogs (21 fecal samples, 16 nasal swabs, and 294 serum samples) and 409 cats (9 fecal samples, 64 nasal swabs, and 336 serum samples) in Shanghai, Guangzhou, Foshan, and Zhengzhou in China. Essential background data on the animals were also documented. The samples were stored at −80°C until processing. Each sample was collected after permission was obtained from the owner, and the procedures met the requirements of the Experimental Animal Welfare Ethics Committee of South China Agricultural University.

### 2.2. Pretreatment of clinical samples

Nasal swabs and fecal samples: The samples were vortexed for 20 s and then centrifuged at 4°C and 12,000 r/min for 10 min, and the supernatant was transferred to a 1.5 mL centrifuge tube. Blood samples were centrifuged at 4°C and 4,000 r/min for 10 min, and the supernatant was transferred to a 1.5 mL centrifuge tube. All samples were stored at −80°C.

### 2.3. Nucleic acid extraction and reverse transcription

To detect CanineCV and the DNA virus associated with it, total viral nucleic acid was extracted from clinical samples. Nucleic acid was extracted using a RaPure Virus DNA/RNA Kit (Magen, Guangzhou, China) according to the manufacturer’s instructions. To detect CanineCV-associated RNA viruses, the extracted viral nucleic acids were reverse-transcribed. cDNA of CanineCV-positive samples was obtained using 5 × ABScript III RT Mix (Abclonal, Wuhan, China) according to the manufacturer’s instructions.

### 2.4. PCR detection of CanineCV and other pathogens

First, CanineCV was detected using primers reported in previous research (q-Rep218F 5′-GTTCGCCGWTGGTGCTT and q-Rep218R 5′-CCCGAGCAGGCTCAAAAT) (Hao et al., 2022). The amplification protocol included predenaturation at 98°C for 1 min, followed by 35 cycles of denaturation at 98°C for 20 s, annealing at 57°C for 30 s, and extension at 72°C for 15 s, with a final extension at 72°C for 5 min. The amplified band of the positive sample was 218 bp.

Other viral pathogens [Canine parvovirus virus 2 (CPV-2), Canine corona virus (CCoV), Canine astrovirus (CaAstV), Canine calicivirus (CaCV), Canine rotavirus (CRV), Canine distemper virus (CDV), Feline calicivirus (FCV) and Feline panleukopenia virus (FPV)]were also detected using specific primers and PCR programs, as previously reported (Gentsch et al., 1992; Jiang et al., 1999; Zhu et al., 2011; Hao et al., 2019; Chang et al., 2021; Zobba et al., 2021; Xiao et al., 2023). All primers (Table 1) were synthesized by Sangon Biotech (Shanghai, China).

**Table 1:** Primers used for PCR detection.

Pathgeon	Primer name	Primer sequence (5′-3′)	PCR products
CPV-2	CPV2-F	AAGACGTGCAAGCGAGTCC	337 bp
	CPV2-R	GAGCGAAGATAAGCAGCGTAA	
CCoV	CCoV-F	AGGAAGGCAACAATCCAATA	477 bp
	CCoV-R	GCCACCTCTGATGGACGA	
CaAstV	CaAstV-F	CAANTCACAACCCAAAACAAA	480 bp
	CaAstV-R	TTTTNACNATCACTGCTAGNG	
CaCV	CaCV-F	GATTACTCCASSTGGGAYTCMAC	319 bp
	CaCV-R	TGACGATTTCATCATCMCCRTA	
CRV	CRV-F	ATTTCGGACCATTTATAACC	876 bp
	CRV-R	TGGCTTCGCCATTTTATAGACA	
CDV	CDV-F	AGATTCAGCCATTTGTAGCCA	794 bp
	CDV-R	GTTGGACTACCTGAGCCCTA	
FCV	FCV-F	AACCTGCGCTAACGTGCT	1940 bp
	FCV-R	TGWATTCCCATGTAGGAGGC	
FPV	FPV-F	AAGACGTGCAAGCGAGTCC	337 bp
	FPV-R	GAGCGAAGATAAGCAGCGTAA	

### 2.5. Amplification and sequencing of the CanineCV complete genome

According to the detected results, CanineCV-positive samples were selected to perform amplification of the full-length CanineCV genome. First, based on rolling circle amplification (RCA), Phi29 DNA Polymerase (Transgen, Beijing, China) was used to amplify the CanineCV genomes in the samples (Rector et al., 2004). Subsequently, one pair of primers was designed to amplify the full genome of CanineCV (CanineCV-F 5′-TCTCGCTCCGCCCGAAGCAC and CanineCV-R 5′-ACGCACCGCCTCCCCTCGCA), and the viral genome was amplified by PCR using ApexHF HS DNA Polymerase (AG, Hunan, China). The 2,123 bp band was purified and cloned into the M5 Hiper pTOPO-Blunt vector (Mei5bio, Beijing, China). The ligation products were transformed into DH5α cells, and positive clones tested by PCR were sent to Sangon Biotech for sequencing. Finally, genome sequences were assembled using SnapGene software (version 4.2.4).

### 2.6. Genetic characterization and phylogenetic analyses

Nucleotide sequences were edited using the BioEdit program and aligned by the ClustalW method. Homology analysis was performed with the DNAStar software package (MegAlign). The obtained complete genomes were aligned with 199 reference sequences of CanineCV and 13 reference sequences of other circoviruses in the GenBank database using the MAFFT algorithm.^1^ A phylogenetic tree was constructed using MEGA (version 7.0.26), and evolutionary distances were computed using the neighbor-joining method (*p*-distance model). Statistical support was provided by 1,000 bootstrap replicates. Other circoviruses were selected as an outgroup to determine the roots of the evolutionary tree.

### 2.7. Recombination analyses

RDP (version 4) was used to analyze the whole genomes of all CanineCV strains that had been aligned with MAFFT, and all sequences were downloaded from GenBank. Recombination events were considered only if they were detected by at least four of seven programs (RDP, GENECONV, Maxchi, Chimera, 3Seq, Bootscan, and SiSscan) with a *p* value <0.05. The recombinant breakpoints were confirmed using SimPlot, with a sliding window of 200 bp (step:10 bp). And phylogenetic analysis was used for further verification.

## 3. Results

### 3.1. Prevalence of CanineCV in dogs and cats

PCR detection showed that the prevalence of CanineCV was 9.06% (30/331, 95% CI = 6.2% ~ 12.7%) in dogs and 3.42% (14/409, 95% CI = 1.9% ~ 5.7%) in cats. The prevalence of CanineCV was 25.45% (14/55, 95% CI = 14.7% ~ 39.0%) among dogs with diarrheic or respiratory symptoms; the prevalence among healthy dogs was 8.7% (6/69, 95% CI = 3.3% ~ 18%). The prevalence of CanineCV was 3.64% (2/55, 95% CI = 0.4% ~ 12.5%) among cats with diarrheic or respiratory symptoms, whereas it was 1.11% (1/90, 95% CI = 0% ~ 6%) among healthy cats (Tables 2, 3). These data show that the prevalence of CanineCV among animals with diarrheic or respiratory symptoms was higher than that among healthy animals, indicating that CanineCV is associated with animal diseases. To elucidate the possibility of a correlation between CanineCV and diarrhea and respiratory disease, the data were processed for calculating the Phi coefficient of association. The correlation coefficient of CanineCV and canine diarrhea and respiratory disease was 0.221. The *p* value of pearson chi-square test was 0.012 (*χ*^2^ = 6.354) and Phi was 0.226 (*p* = 0.012), respectively. The result suggested a positive association between CanineCV and canine diarrhea and respiratory disease. The correlation coefficient of CanineCV and feline diarrhea and respiratory disease was 0.086. The *p* value of pearson chi-square test was 0.3 (*χ*^2^ = 1.074) and Phi was 0.086 (*p* = 0.3), respectively. The result suggested no association between CanineCV and feline diarrhea and respiratory disease.

**Table 2:** Positive detection rates of CanineCV in different samples.

Species	Health status	Number of positive samples	Number of samples	Positive detection rate (%)	95% CI	Correlation coefficient	Pearson chi-square test	Phi
Dog	Healthy	6	69	8.7	3.3% ~ 18%	0.221	0.012 (χ^2^ = 6.354)	0.226 (*p* = 0.012)
	Sick	14	55	25.45	14.7% ~ 39.0%			
Cat	Healthy	1	90	1.11	0% ~ 6%	0.086	0.3 (χ^2^ = 1.074)	0.086 (*p* = 0.3)
	Sick	2	55	3.64	0.4% ~ 12.5%			

**Table 3:** General information and detection results for the positive samples in this study.

Sample name	Location	Species	Sample type	Other pathogens detected
1^*^	Shanghai	Dog	Feces	CPV-2
2^*^	Guangzhou	Dog	Feces	CPV-2
3	Guangzhou	Dog	Serum	-
4	Guangzhou	Dog	Serum	-
5	Guangzhou	Dog	Serum	-
6	Guangzhou	Dog	Serum	-
7^*^	Guangzhou	Dog	Serum	-
8	Guangzhou	Dog	Serum	-
9	Guangzhou	Dog	Serum	-
10^*^	Guangzhou	Dog	Serum	-
11	Guangzhou	Dog	Serum	-
12	Guangzhou	Dog	Serum	CPV-2
13	Guangzhou	Dog	Serum	-
14	Guangzhou	Dog	Serum	-
15^*^	Guangzhou	Dog	Serum	-
16	Guangzhou	Dog	Serum	-
17	Zhengzhou	Dog	Feces	CCoV+CaCV
18	Zhengzhou	Dog	Feces	CaCV
19	Zhengzhou	Dog	Feces	CPV-2 + CCoV+CaCV
20	Zhengzhou	Dog	Feces	CCoV+CaCV
21	Guangzhou	Dog	Serum	CPV-2
22	Guangzhou	Dog	Serum	CPV-2
23^*^	Guangzhou	Dog	Serum	CPV-2
24^*^	Guangzhou	Dog	Serum	CPV-2
25	Zhengzhou	Dog	Feces	CPV-2 + CaCV
26	Zhengzhou	Dog	Feces	-
27	Zhengzhou	Dog	Feces	CPV-2 + CCoV
28	Guangzhou	Dog	Serum	-
29^*^	Foshan	Dog	Feces	-
30	Guangzhou	Dog	Nasal swab	CCoV
31	Guangzhou	Cat	Serum	-
32	Guangzhou	Cat	Serum	-
33	Guangzhou	Cat	Serum	-
34	Guangzhou	Cat	Serum	FPV
35	Guangzhou	Cat	Serum	-
36	Guangzhou	Cat	Serum	FPV
37	Guangzhou	Cat	Serum	-
38	Guangzhou	Cat	Serum	FPV
39	Guangzhou	Cat	Serum	-
40	Guangzhou	Cat	Serum	-
41	Guangzhou	Cat	Serum	FPV
42	Guangzhou	Cat	Serum	FPV
43	Foshan	Cat	Nasal swab	-
44^*^	Guangzhou	Cat	Nasal swab	-

“-” denotes negative; “*” denotes samples that successfully amplified the full genome sequence; canine coronavirus (CCoV), canine parvovirus 2 (CPV-2), canine calicivirus (CaCV), feline panleukopenia virus (FPV).

Of all positive samples, 46.67% (14/30) dog-positive samples were coinfected with other canine pathogens (CPV-2 or CCoV or CaCV); 35.71% (5/14) cat-positive samples were coinfected with FPV.

### 3.2. Whole genome of CanineCV

Fifteen full genomes of CanineCV strains were obtained, as amplified from 9 positive samples: isolates SH1, SH2, SH3, GZ1, GZ2, GZ3, GZ4, GZ5, GZ6, GZ7, GZ8, GZ9, FS1, FS2, and FS3 (GenBank ID OP575971-OP575985), with GZ9 being amplified from a cat sample named 44.

All 15 CanineCV genomes were 2063 nt in length, consistent with most CanineCV strains in the GenBank database. Sequence analysis revealed a nucleotide composition of 52 ~ 53% GC and 47 ~ 48% AT in these CanineCV strains. Nucleic acid Homology analysis showed that the 15 CanineCV strains shared 86.6% ~ 100% identity and 82.1% ~ 99.3% identity with published sequences in GenBank (Table 4). Moreover, two main ORFs indicated that Rep and Cap had a high degree of variation at both the nt and aa levels.

**Table 4:** Homology analysis of CanineCV.

Homology analysis	Whole genome (%)	Rep (%)		Cap (%)	
	nt	nt	aa	nt	aa
CanineCV obtained in this study	86.6 ~ 100	85.4 ~ 100	92.9 ~ 100	84.3 ~ 100	93.3 ~ 100
CanineCV obtained in this study with published sequences in the GenBank database	82.1 ~ 99.3	79.6 ~ 99.1	85.9 ~ 99	79.2 ~ 99.4	85.6 ~ 100

### 3.3. Phylogenetic analysis

**Figure 1:**
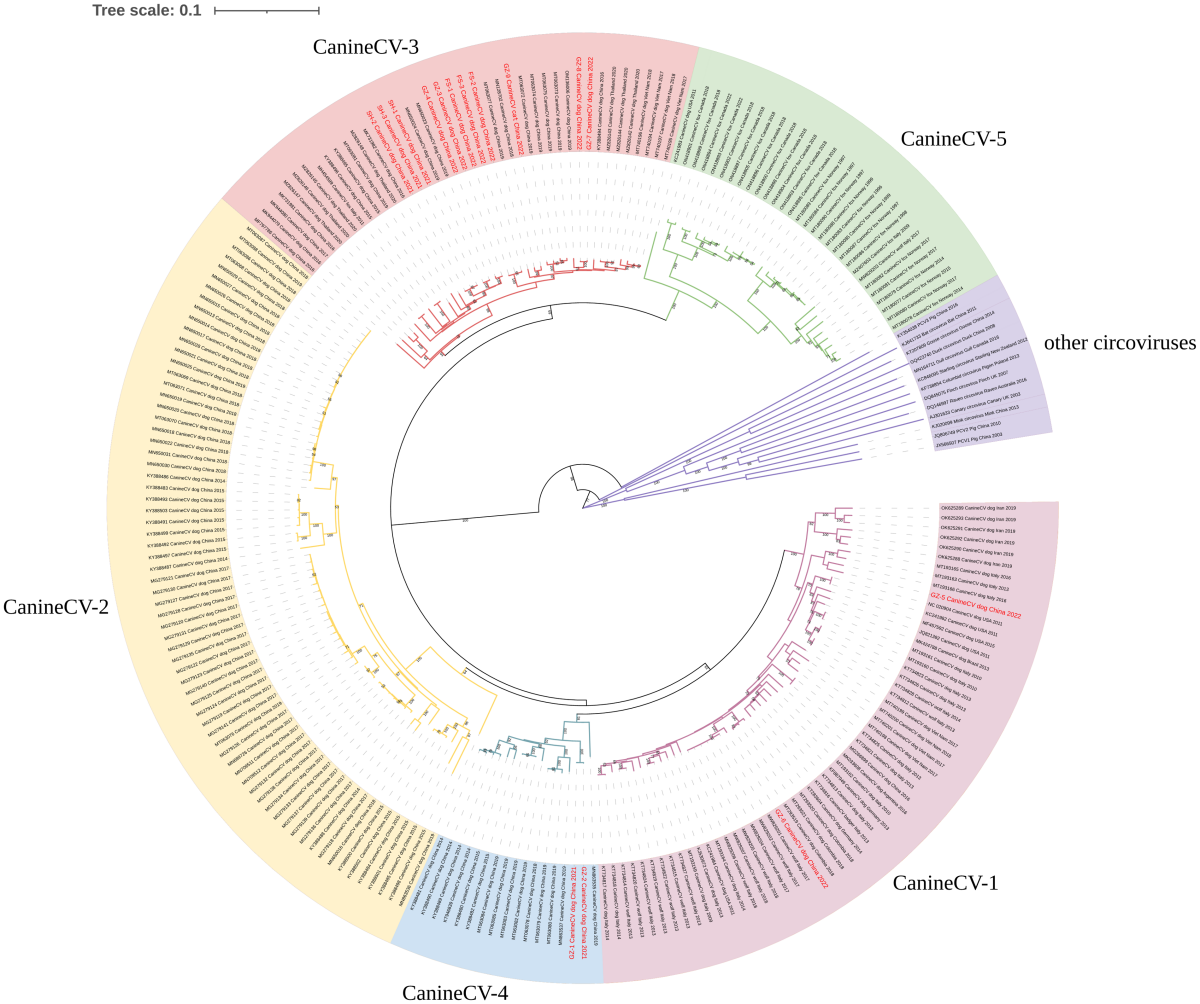
Neighbor-joining phylogenetic tree based on the full genome of CanineCV. Statistical support was provided by bootstrapping with 1,000 replicates. The red font indicates the strains obtained in this study.

The NJ phylogenetic tree was constructed based on CanineCV whole genomes. Phylogenetic analysis showed that all CanineCV strains segregated into 5 clades, corresponding to 5 genotypes (Figure 1). The CanineCV sequences identified in this study were divided into 3 genotypes (CanineCV-1, CanineCV-3, and CanineCV-4). Among, the first cat-derived CanineCV belongs to genotype 3 (Figure 1).

### 3.4. Recombination analyses

**Figure 2:**
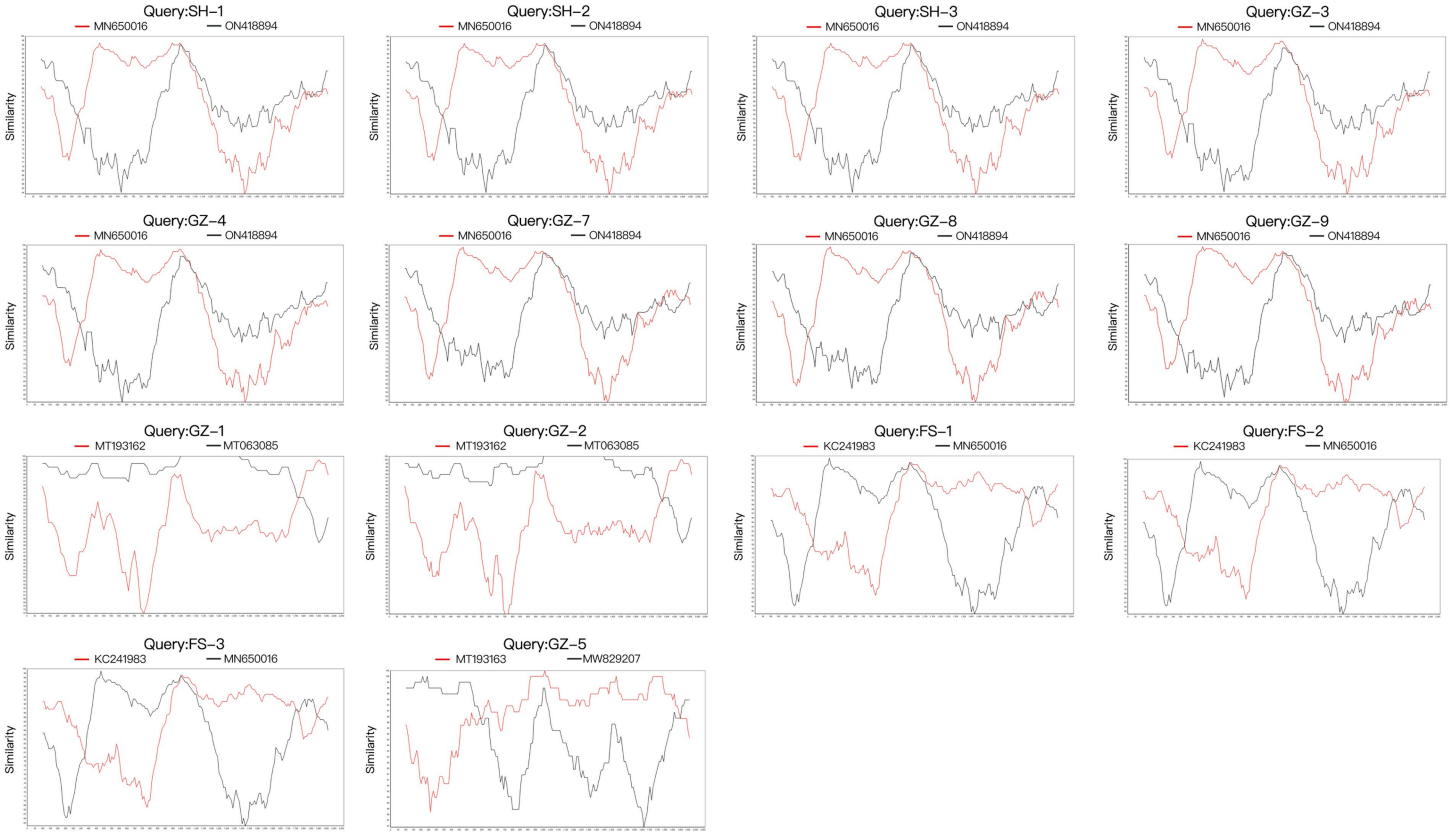
Recombination analysis of the 15 full genomes of CanineCV strains using SimPlot v3.5.1 with a window and step sizes of 200 nt and 10 nt, respectively.

**Figure 3:**
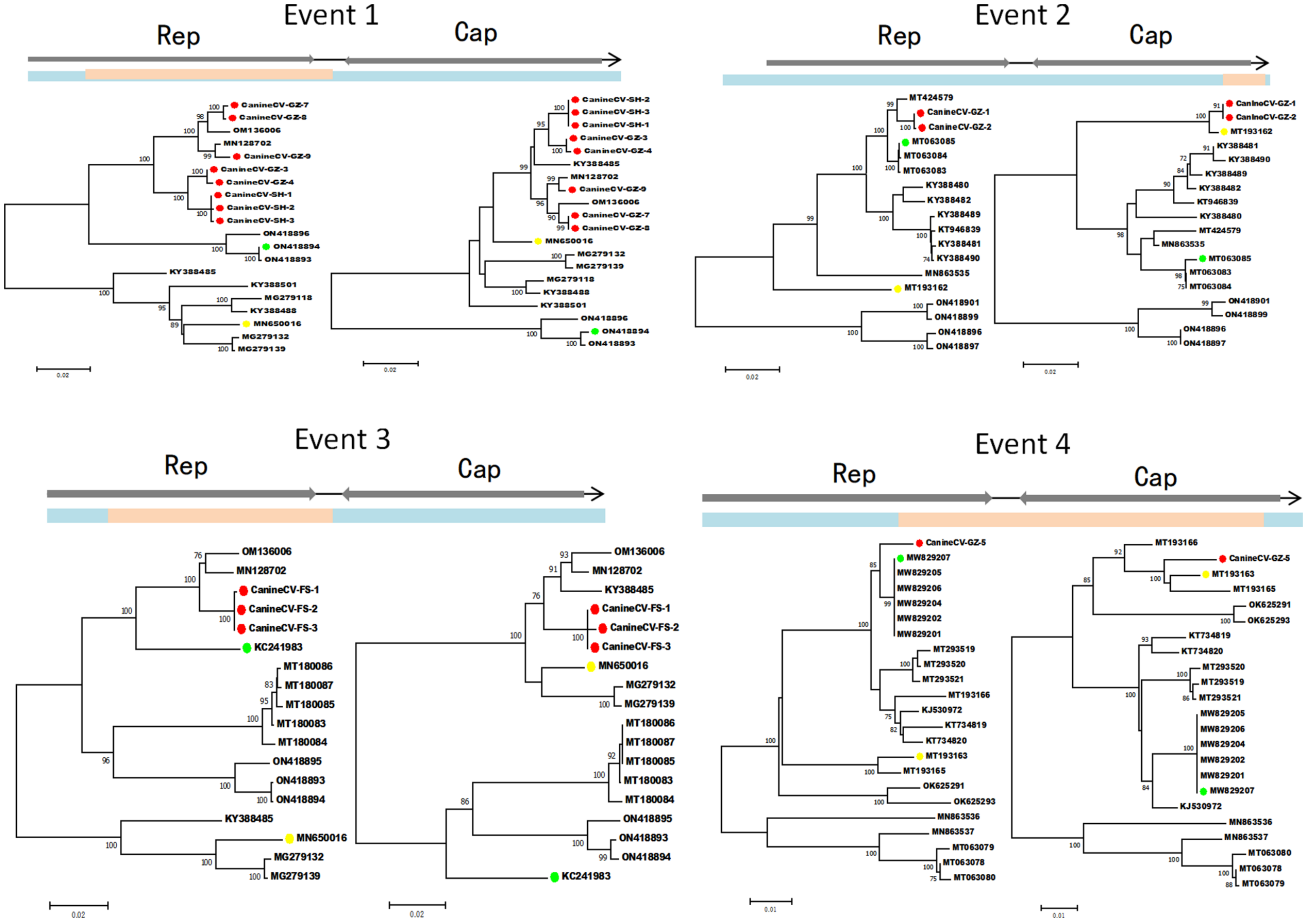
Phylogenetic trees illustrate the potential recombination events detected in this study. Blue indicates the sequence region derived from the major parent; pink indicates the sequence region derived from the minor parent. The dots marked in green, yellow, and red indicate the potential major parent, minor parent, and recombinant sequences, respectively. The phylogenetic trees were reconstructed using the neighbor-joining method. Statistical support was provided by bootstrapping with 1,000 replicates. The numbers (>70) above branches indicate percent bootstrap values.

Four potential recombination events with a transferred fragment ranging in size from 11.3% ~ 61.6% of the genome were detected using RDP4 software (Table 5), and the results were further confirmed using Simplot (Figure 2) and phylogenetic analyses (Figure 3). Notably, recombination events occurred not only in the 2 ORF genes but also in other parts of the genome. Moreover, events 1, 2, and 3 were intergenotypic recombination events, whereas event 4 was a recombination event that occurred within genotype 3. Interestingly, the major parent and minor parent of recombination event 4 were isolated from Italian wolf and dog, respectively. In addition, the major parents of other recombination events were isolated from dogs in Canada, China, and the United States. The minor parents of other recombination events were isolated from dogs in China and Italy.

**Table 5:** Information on recombination events detected using the RDP (R), GENECONV (G), Maxchi (M), Chimera (C), 3Seq (Q), Bootscan (B), and SiSscan (S) methods implemented in the computer program RDP4.

Event	Recombination sequence	Major parent	Minor parent	Position (in alignment)	Detection method (*p* < 0.05)
1	SH-1, SH-2, SH-3, GZ-3, GZ-4, GZ-7, GZ-8, GZ-9	ON418894 (Canada)	MN650016 (China)	343–1,062	RGBMCST
2	GZ-1, GZ-2	MT063085 (China)	MT193162 (Italy)	1794–2028	RGBMCST
3	FS-1, FS-2, FS-3	KC241983 (United States)	MN650016 (China)	354–1,062	RGBMCST
4	GZ-5	MW829207 (Italy)	MT193163 (Italy)	616–1887	RMST

## 4. Discussion

CanineCV has been discovered in dogs and some carnivorous wild animals. In this study, the prevalence of CanineCV was 25.45% (14/55, 95% CI = 14.7% ~ 39.0%) among dogs with diarrheic or respiratory symptoms; the prevalence among healthy dogs was 8.7% (6/69, 95% CI = 3.3% ~ 18%). The high prevalence of CanineCV among dogs is consistent with previous reports from Iran and Thailand (Turan and Işıdan, 2020; Beikpour et al., 2022). Our previous study described the first identification and full genomic characterization of feline stool-associated circular DNA virus in cats in China (Hao et al., 2021). Due to the close contact between cats and dogs, this study also investigated presence of CanineCV in cats. The prevalence of CanineCV in cats, 2.07%, was identified for the first time in this study. These data remind us of the importance of paying close attention to the prevalence of CanineCV in dogs and cats.

The CanineCV prevalence in animals with diarrhea or respiratory symptoms was higher than that in healthy animals, which is consistent with previous studies (Li et al., 2013; Decaro et al., 2014; Hsu et al., 2016; Anderson et al., 2017; Cruz et al., 2020). Moreover, 43.18% (19/44) of positive samples showed coinfection with other pathogens, which is also consistent with previous studies conducted in Italy and China (Hsu et al., 2016; Beikpour et al., 2022). Our previous study showed that CanineCV occurs in coinfections with other viruses, and immunosuppression may occur, aggravating clinical symptoms and mortality (Hao et al., 2022). Therefore, CanineCV detection in dogs and cats with diarrhea or respiratory symptoms is necessary.

CanineCV is divided into five genotypes (CanineCV-1, −2, −3, −4, and − 5) based on the whole genome (Urbani et al., 2021). The CanineCV-1 genotype is mainly reported in the USA, Europe, and Asia. The CanineCV-2, −3, and − 4 genotypes are mainly found in Asia (Niu et al., 2020; Urbani et al., 2021). Additionally, the CanineCV-5 genotype has been detected in Europe and North America (Urbani et al., 2021). This study obtained 15 genomes of CanineCV, and homology analysis demonstrated that all these strains shared a sequence identity ranging from 86.6 to 100%. The strains from Shanghai were genotype 3, whereas the strains from Guangdong were genotypes 1, 3, and 4. Notably, the strain from a cat was classified as genotype 3. This result provides evidence for the prevalence of at least 3 genotypes of CanineCV in Guangdong, indicating the complex circulation of CanineCV among dogs and cats.

Several circoviruses, including CanineCV, have undergone genetic recombination during evolution (Piewbang et al., 2018; Stenzel et al., 2018; Sun et al., 2019; Niu et al., 2020; Dankaona et al., 2022; Yuan et al., 2022). Four potential recombination events were detected in this study, including intragenotype and intergenotype recombination. Moreover, recombination events occurred not only in the 2 ORF genes but also in other parts of the genome, which is consistent with previous results (Piewbang et al., 2018; Sun et al., 2019; Tuong et al., 2021). More importantly, the major and minor parents in these 4 recombination events were not only from China but also from Europe and North America. Therefore, it is necessary to closely monitor the genomic recombination of CanineCV.

In conclusion, this study is the first to provide evidence of CanineCV infection in cats and successfully obtained the first whole genome of cat-derived CanineCV. The complex circulation and high prevalence of CanineCV among dogs and cats emphasize the importance of continuous monitoring of this virus in various animal species.

## Data availability statement

The datasets presented in this study can be found in online repositories. The name of the repository/repositories can be found at: https://www.ncbi.nlm.nih.gov/genbank/. Accession numbers are OP575971-OP575985.

## Ethics statement

The animal studies were approved by the Experimental Animal Welfare Ethics Committee of South China Agricultural University. The studies were conducted in accordance with the local legislation and institutional requirements. Written informed consent was obtained from the owners for the participation of their animals in this study.

## Author contributions

XX and YL participated in writing original draft and writing–review and editing. XX, YL, FX, and XH performed the experiments. SL and PZ contributed to conceptualization, methodology, validation, resources, writing–review and editing, supervision, and funding acquisition. All authors read and approved the final manuscript.

## Funding

This work was supported by the Natural Science Foundation Guangdong province (2023A1515012171 and 2022A1515010733) and Guangzhou Municipal Science and Technology Bureau (SL2022A04J00674).

## Conflict of interest

The authors declare that the research was conducted in the absence of any commercial or financial relationships that could be construed as a potential conflict of interest.

## Publisher’s note

All claims expressed in this article are solely those of the authors and do not necessarily represent those of their affiliated organizations, or those of the publisher, the editors and the reviewers. Any product that may be evaluated in this article, or claim that may be made by its manufacturer, is not guaranteed or endorsed by the publisher.

## Footnotes

^1^ https://mafft.cbrc.jp/alignment/software/
